# Renal denervation by radiofrequency in patients with hypertension: systematic review and meta-analysis

**DOI:** 10.1590/1806-9282.2023D704

**Published:** 2024-05-13

**Authors:** Antonio Silvinato, Idevaldo Floriano, Wanderley Marques Bernardo

**Affiliations:** 1 Evidence-Based Medicine, Brazilian Medical Association – São Paulo (SP), Brazil.; 2 Universidade de São Paulo, Faculty of Medicine – São Paulo (SP), Brazil

## Abstract

The Guidelines Project, which is an initiative of the Brazilian Medical Association, aims to combine information from the medical field to standardize how to conduct and assist in the reasoning and decision-making of doctors. The information provided by this project must be critically evaluated by the physician responsible for the conduct that will be adopted, depending on the conditions and the clinical condition of each patient.

## INTRODUCTION

Hypertension is a significant risk factor for cardiovascular morbidity and mortality^1^. Despite a wide array of pharmacological treatment options, many patients remain uncontrolled^2^. Medical inertia and patient non-adherence to medications are the main reasons for this lack of control. Hyperactivity of the sympathetic nervous system plays a crucial role in resistant hypertension. The sympathetic renal nerves primarily originate in the celiac and aortorenal ganglia around the aorta. The renal nerves run in the adventitia and perivascular adipose tissue around the renal arteries. At the renal level, the sympathetic efferent pathway (brain → kidney) directed to the kidneys results in increased production of noradrenaline, causing renal vasoconstriction and the release of renin, which in turn induces sodium retention. On the contrary, the afferent sympathetic fibers (kidney → brain) transmit signals to the brain, stimulating central sympathetic activity and contributing to neurogenic hypertension^3^. It is common to find an increase in sympathetic system activity in hypertension^4^, especially in the presence of obesity^5^. In the past decade, renal denervation (RDN) has emerged as a treatment option for arterial hypertension. It is performed through the percutaneous insertion of the device catheter into the femoral artery, which is then advanced into the main renal arteries under fluoroscopic guidance^6^. RDN is a catheter-based ablation of the afferent and efferent sympathetic nerves within the wall of the renal arteries. Generally, the delivery of energy through radiofrequency or ultrasound heats up the surrounding adipose tissue of the renal arteries, where the renal nerves are located. Therefore, the renal nerves are destroyed as a result of a thermal injury^3^. The complete report of a trial [SPYRAL HTN-ON MED, 2023] (which constitutes 46% of the total data from second-generation placebo-controlled trials) has emerged recently. Therefore, we conducted an updated meta-analysis of RDN, by radiofrequency (RF-RDN), for hypertension, including the entirety of the data from second-generation randomized placebo-controlled trials, which are currently available.

## OBJECTIVE

The objective of this study was to evaluate the benefits and harms of RF-RDN for the treatment of patients with uncontrolled hypertension, in the presence or absence of antihypertensive medications.

## METHODOLOGY

This systematic review followed the Preferred Reporting Items for Systematic Reviews and Meta-analyses (PRISMA)^7^ and is supported by scientific information obtained through a systematic review of the literature (published).

### Eligibility criteria

The eligibility criteria express the specific elements to answer the clinical question of this evaluation (objective). Considering the various arguments against combining first- and second-generation RDN trials in a single meta-analysis, we chose to include only "second-generation RF-RDN trials." These stand out not only for the strict control of medication intake but also for improvements in all other practical methods. This includes blood pressure (BP) measurements, patient selection, and the execution of the RF-RDN procedure itself. In addition, "second-generation trials" feature new catheter designs and a greater number of ablations in the arteries, thus being enhanced and distinct compared with "first-generation trials."

### Criteria for study inclusion:

- Patients: Individuals with uncontrolled hypertension, whether or not they are using antihypertensive medications.
- Intervention: Renal sympathetic denervation through radiofrequency.
- Comparison: Placebo.
- Outcomes: Clinically relevant efficacy, and in the absence of such data, intermediate outcomes including reduction in BP (mmHg) and safety.
- Study design: Double-blind randomized controlled parallel trials.
- Language: No restrictions.
- Consultation period: No restrictions.
- Full text availability: Required.

Systematic reviews with or without meta-analysis, narrative reviews, observational and/or case series, first-generation RF-RDN trials or absence of extractable data (absolute numbers and/or averages), and Phase 2 study were excluded from this study.

**Search for Evidence** will be carried out in the virtual scientific information base Medline using the search strategy: #1 ((Blood Pressure OR Hypertension) AND Kidney AND (Catheter Ablation OR Catheters OR Catheterization)), #2 ((Blood Pressure OR Hypertension) AND (Kidney OR Renal Artery) AND (Sympathectomy* OR Denervation OR Endovascular Procedures)), ((#1) OR (#2)) AND (Random*); CENTRAL / Cochrane: ((Blood Pressure OR Hypertension) AND (renal denervation)); LILACS: Hypertension AND Renal AND Denervation AND (type_of_study:("clinical_trials")); and ClinicalTrials.gov: Hypertension AND Renal AND Denervation, Study Typ=Interventional (Clinical Trial). Additional manual searches were conducted in the reference list of the included studies and other relevant sources. The search in these databases was carried out until January 2024.

### Study selection process and data extraction

The evidence retrieved from the consulted databases is initially selected based on the title and abstract, aiming to meet the eligibility criteria. The related studies in this first selection then have their full texts accessed to confirm their eligibility. The process of retrieving the studies, as well as the evaluation of the obtained titles and abstracts, was conducted by two researchers skilled in the development of systematic reviews (A.S. and I.F.) independently and blinded, following the inclusion and exclusion criteria. Subsequently, the selected articles were critically evaluated to be included or not in the review. When there was disagreement about the selection of studies among the investigators, a third reviewer was consulted (W.M.B.).

In the selected studies, we will extract the following data: author's name and year of publication, the studied population, intervention and comparison methods, and follow-up duration. For relevant outcomes, data extraction may include absolute event numbers or means and/or medians, along with corresponding standard deviations (SDs) or 95% confidence intervals (95%CIs), depending on the type of outcome.

### Risk of bias and quality of evidence

Two independent reviewers assessed the risk of bias in the included studies using the items from the Cochrane Risk of Bias Tool for randomized trials (RoB 2)^8^, supplemented with other fundamental elements, and expressed as high, moderate, and low. The levels of evidence will be extrapolated from the risk of bias obtained from the study/studies (if there is no meta-analysis) using the terminology of the Grading of Recommendations Assessment, Development, and Evaluation (GRADE)^9^ as very low, low, and high, and through the GRADEpro software^10^ (if there is a meta-analysis) as very low, low, moderate, and high. Two reviewers assessed the risk of bias, inconsistency, indirect evidence, imprecision, and publication bias for all reported outcomes.

### Method of analysis and synthesis of results

The data were analyzed following the intention-to-treat (ITT) principle, and each trial included the most recent follow-up data. Categorical outcomes were reported as the risk difference (RD) between the intervention and control groups. If the RD was statistically significant (95% confidence), it would be presented with the 95%CI and the number needed to treat (NNT) or to produce harm (NNH).

For continuous measures, results were presented as mean differences (MDs) or standardized mean differences (SMDs) when different scales were reported, accompanied by corresponding 95%CIs. In instances where multiple studies with common outcomes were included, meta-analysis would be conducted using the Review Manager 5.4 software (The Nordic Cochrane Centre, The Cochrane Collaboration)^11^. The overall difference in risk or mean, along with 95%CIs, served as the conclusive measure supporting the synthesis of evidence, addressing the clinical question (objective) of this assessment.

In cases where SD information was unavailable, we calculated SD from the sample size using either the standard error (SE) or the 95%CI. The estimation of combined effect sizes was carried out using either a fixed- or random-effect model after evaluating the results for heterogeneity. Statistical heterogeneity was assessed using the I^2^ metric, which measures the percentage of variation related to heterogeneity between studies rather than randomness^12^.

### Evidence synthesis and conclusion

The synthesis of evidence directly present results from the analyses, carefully evaluating the benefits, harms, and absence of differences between the use of RF-RDN through parallel comparison with a placebo. Conclusions were primarily drawn from evidence of at least moderate quality, considering the presence of an effect, whether it was beneficial or harmful, and an overall favorable balance between benefits and harms. This was particularly crucial in patients with difficult-to-control or genuinely resistant hypertension.

## RESULTS

In the quest for evidence regarding the use of RDN, we retrieved 943, 621, 486, and 146 studies from the MEDLINE, CENTRAL, LILACS, and CT.gov databases, respectively. No studies were obtained through manual and/or gray searches. Following the removal of duplicates and exclusion based on title and/or abstract, 20 studies remained, aligning with the previously established eligibility criteria (methodology). These 20 studies were further chosen for access to their full texts.

**Figure 1:**
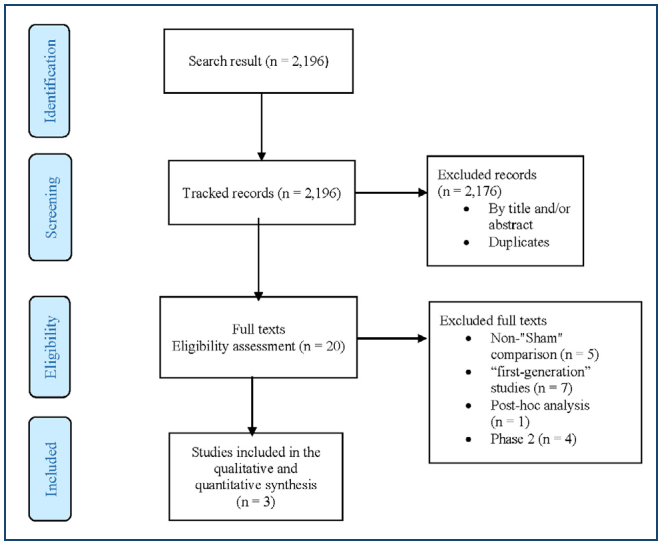
Flow diagram representing the study selection process.

Upon a comprehensive review of the full texts, three randomized controlled trials conducted in parallel with a placebo^13-15^ were included to substantiate the conclusions of this assessment. The exclusion of the other 17 studies was attributed to reasons such as the absence of a comparison of RF-RDN with Sham, involvement in "first-generation" studies, post-hoc analysis, or being classified as a Phase 2 study (refer to Figure 1 for details). The references and reasons for the exclusion of these studies are available in the "References" section. The flow diagram in Figure 1 illustrates the sequence from retrieval to the selection of evidence supporting this assessment.

*From:* Moher D, Liberati A, Tetzlaff J, Altman DG, The PRISMA Group (2009). *P*referred *R*eporting *I*tems for *S*ystematic Reviews and *M*eta-*A*nalyses: The PRISMA Statement. PLoS Med 6(7): e1000097. doi:10.1371/journal. pmed1000097

Three "second-generation trials" that met the inclusion and exclusion criteria were identified^13-15^. The main baseline characteristics and details of each trial are outlined in Table 1 (ANNEXES). These trials collectively involved 719 participants, with 406 randomized to RF-RDN and 313 to the control group.

**Table 1:** Key patient baseline characteristics and details of each trial.

First Author/ Trial, Year (Ref. No.)	Patients (N)	Intervention (N)	Control (N)	Denervation Method	Outcomes	Follow-Up Duration (Months)	Participating Centers
Böhm M, et al. SPYRAL HTN-OFF MED Pivotal, 2020 (13)	Untreated patients over the 3–4 weeks before randomization. Clinic BP 150–179/≥90 mmHg and average 24-h ambulatory BP 140–16 mmHg. Mean age: 53 years. (N = 331; 80 patients from randomized Pilot trial and 251 patients from randomized pivotal trial)	RF-RDN (N = 166)	SHAM: Renal angiography (N = 165)	Radiofrequency technique (Multielectrode Symplicity SpiralTM ®)	Primary end point: change in mean 24-h systolic blood pressure Secondary end point: change in office SBP; changes in morning and nighttime BP Adverse events	3	44 centers in Australia, Austria, Canada, Germany, Greece, Ireland, UK, and USA
Kandzari DE, et al. SPYRAL HTN-ON MED, 2023 (14)	Patients with 1–3 drugs from ≥6 weeks, uncontrolled (clinic BP 150–180/≥90 mmHg, 24-h BP 140–170 mmHg). Mean age: 53-55 years. (N = 337; 80 patients from randomized Pilot trial and 257 patients from randomized Expansion trial).	RF-RDN (N = 206)	SHAM: Renal angiography (N = 131)	Radiofrequency technique (Multielectrode Symplicity SpiralTM ®)	Primary end point: change in ambulatory blood pressure Secondary end point: office BP; changes in morning (daytime) and night-time BP Adverse Events	6	56 clinical centers worldwide (USA, Germany, Japan, UK, Australia, Austria, and Greece)
Weber MA, et al. REDUCE HTN: REINFORCE, 2020 (15)	Patients with office SBP of 150 to 180 mmHg and average 24-h ambulatory SBP of 135 to 170 mmHg after medication washout. Mean age: 58 years	RF-RDN (N = 34) 8 weeks: no antihypertensive medications (unless rescue) 8 weeks to 6 months: add medication if office SBP ≥140 mmHg	SHAM: Renal Angiography (N = 17) 8 weeks: no antihypertensive medications (unless rescue) 8 weeks to 6 months: add medication if office SBP ≥140 mmHg	Radiofrequency technique (Vessix Renal Denervation® system transmits radiofrequency energy via bipolar electrodes).	Primary end point: Mean reduction in average 24-h ambulatory BP. Secondary end point: Daytime ambulatory Office BP Adverse events	8 weeks 6 months: 12 months	12 centers in USA

Radiofrequency (RF); Renal denervation (RDN); Blood pressure (BP); systolic blood pressure (SBP); office blood pressure (OBP).

### Risk of bias in studies

Regarding the risk of bias in the three included randomized clinical trials (RCTs)^13-15^, one did not present an ITT analysis^13^, and another terminated early with 51 patients included out of an expected total of 93, without performing an ITT analysis^14^. The risk of bias assessment for each individual study, conducted using the RoB 2 tool^8^ supplemented with other essential elements, is provided in Table 2.

**Table 2:** Risk of bias in studies.

First Author/Year (Ref. #)	Randomization	Blind allocation	Double-blind		Outcome researcher blind	Losses		Prognostic characteristics	Appropriate outcomes	Intention-to-treat analysis	Sample size calculation	Early interruption	Risk of Bias
Böhm M, 2020 13													High
Kandzari DE, 2023 14													Low
Weber MA, 2020 14													High
LEGEND	HIGH RISK			NOT INFORMED			LOW RISK						

### Outcomes

#### Efficacy

The data extracted from the three RCTs included changes (MDs [±SD]) in comparing BPs at follow-up time and baseline. These results facilitated the calculation of the MD (95%CI) in meta-analyses between the intervention (RF-RDN) and control (SHAM). Relevant clinical outcomes, such as hypertensive crises and strokes, were incorporated into adverse events, with a maximum follow-up of 6 months. The levels of evidence, as per the GRADE system, for each outcome can be found in Table 3 (ANNEXES).

**Table 3:** Levels of evidence – GRADE System. Summary of results: RF-RDN compared with SHAM in blood pressure change for hypertension Patient or population: HYPERTENSION Context: Efficacy and safety Intervention: RF-RDN Comparison: SHAM.

Outcomes Number of participants (studies)	Mean Difference	Certainty
24-h ambulatory systolic blood pressure – 2–3 months No. of participants: 719 (3 RCTs)	MD **2.5 lower**(4 lower to 1 lower)	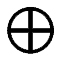 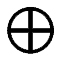 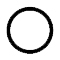 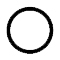 Low a,b
24-h ambulatory systolic blood pressure – 6 months No. of participants: 388 (2 RCTs)	MD **2.33 lower** (4.54 lower to 0.12 lower)	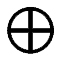 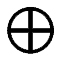 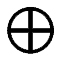 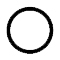 Moderate c
24-h ambulatory diastolic blood pressure – 2–3 months No. of participants: 719 (3 RCTs)	MD **2.18 lower**(3.17 lower to 1.2 lower)	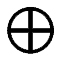 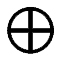 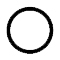 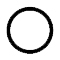 Low a,b
24-h ambulatory diastolic blood pressure – 6 months No. of participants: 388 (2 RCTs)	MD **1.07 lower**(2.66 lower to 0.53 higher)	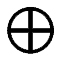 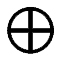 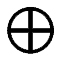 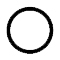 Moderate c
In-office systolic blood pressure – 2–3 months No. of participants: 719 (3 RCTs)	MD **4.48 lower**(6.48 lower to 2.49 lower)	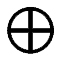 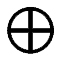 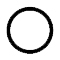 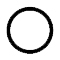 Low a,b
In-office systolic blood pressure – 6 months No. of participants: 388 (2 RCTs)	MD **5.7 lower**(8.45 lower to 2.96 lower)	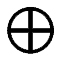 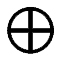 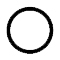 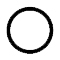 Low b,c
In-office diastolic blood pressure – 2–3 months No. of participants: 719 (3 RCTs)	MD **2.63 lower**(3.86 lower to 1.4 lower)	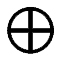 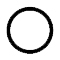 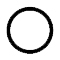 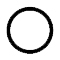 Low a,b
In-office diastolic blood pressure – 6 months No. of participants: 388 (2 RCTs)	MD **2.03 lower**(3.84 lower to 0.22 lower)	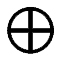 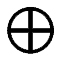 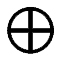 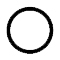 Moderate c

Explanations:

RCTs: randomized controlled trials.

a Two studies were included, with a high risk of bias (no intention-to-treat analysis in both, and early termination in one), and one with low risk.

b Substantial heterogeneity.

c Two studies were included, with one not undergoing intention-to-treat analysis and experiencing early termination.

**Table:** 

Outcome No. of participants (studies)	Relative effect(95%CI)	Potential absolute effects (95%CI)			Certainty
		RF-RDN	SHAM	Difference	
Serious adverse events No. of participants: 719 (3 RCTs)	**RR 0.87**(0.21–3.52)	1.0%	**0.8%**(0.2–3.4)	0.1% fewer (0.8 fewer to 2.4 more)	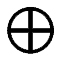 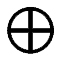 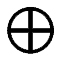 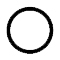 Moderate ^a^

Explanations:

* The risk in the intervention group (and its 95% confidence interval) is based on the assumed risk in the comparator group and the relative effect of the intervention (and its 95%CI). CI: confidence interval; MD: mean difference; RR: risk ratio.

a. Two studies were included, with a high risk of bias (no intention-to-treat analysis in both, and early termination in one), and one with low risk. **Table:** 

**GRADE Working Group grades of evidence**
**High certainty:** We are very confident that the true effect lies close to that of the estimate of the effect.
**Moderate certainty:** We are moderately confident in the effect estimate: The true effect is likely to be close to the estimate of the effect, but there is a possibility that it is substantially different.
**Low certainty:** Our confidence in the effect estimate is limited: The true effect may be substantially different from the estimate of the effect.
**Very low certainty:** We have very little confidence in the effect estimate: The true effect is likely to be substantially different from the estimate of effect.

**Figure 2:**
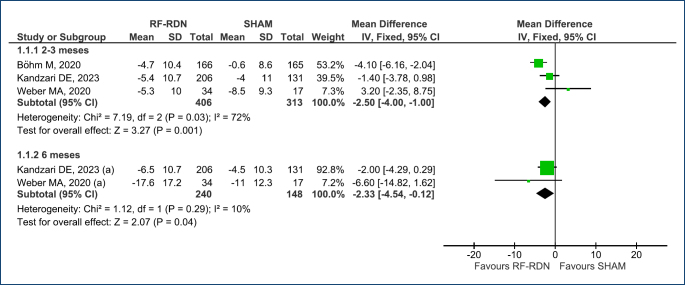
Forest plot of comparison: 1 RF-RDN versus SHAM, outcome: 1.1 Change in 24-h ambulatory systolic BP.

**Figure 3:**
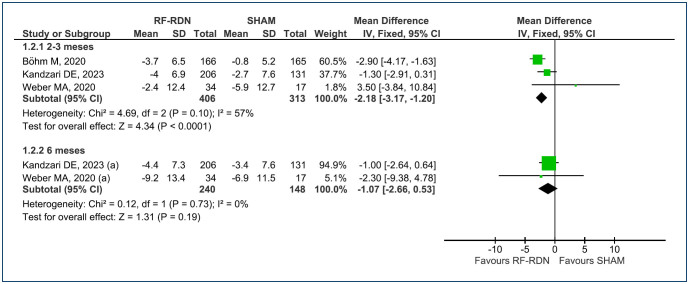
Forest plot of comparison: 1 RF-RDN versus SHAM, outcome: 1.2 Change in 24-h ambulatory diastolic BP.

The mean change in ambulatory (24 h) systolic and diastolic BP at 2–3-month follow-up was assessed in three RCTs^13-15^ (total of 719 patients). Compared with SHAM, the RF-RDN procedure demonstrated a reduction of -2.50 mmHg [95%CI (-4.00, -1.00); p<0.001; I^2^=72%] and -2.18 mmHg [95%CI (-3.17, -1.20); p<0.0001; I^2^=58%], respectively (Figures 2 and 3). According to the GRADE system, the level of evidence is considered low.

For the change in mean ambulatory (24 h) systolic and diastolic BP at 6-month follow-up, data were available from two RCTs^14,15^ (a total of 388 patients). Compared with SHAM, the RF-RDN procedure exhibited a reduction of -2.33 mmHg [95%CI (-4.54, -0.12); p<0.04; I^2^=10%] in systolic BP and no significant difference in diastolic BP [-1.07 mmHg [95%CI (-2.66, 0.53); p<0.19; I^2^=0%] (Figures 2 and 3). Based on the GRADE system, the level of evidence is moderate.

**Figure 4:**
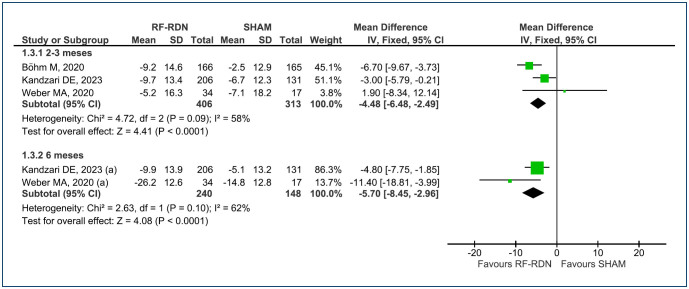
Forest plot of comparison: 1 RF-RDN versus SHAM, outcome: 1.3 Change in office systolic BP.

**Figure 5:**
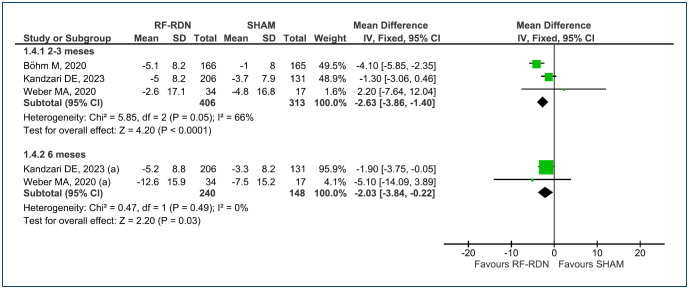
Forest plot of comparison: 1 RF-RDN versus SHAM, outcome: 1.4 Change in office diastolic blood pressure.

The mean change in office systolic and diastolic BP at 2–3-month follow-up was analyzed across three RCTs^13-15^ (a total of 719 patients). When compared with SHAM, the RF-RDN procedure demonstrated a reduction of -4.48 mmHg [95%CI (-6.48, -2.49); p<0.0001; I^2^=58%] and -2.63 mmHg [95%CI (-3.86, -1.40); p<0.0001; I^2^=66%], respectively (Figures 4 and 5). The level of evidence is considered low.

For the mean change in office systolic and diastolic BP at 6-month follow-up, data were available from two RCTs^14,15^ (total of 388 patients). In comparison with SHAM, the RF-RDN procedure exhibited a reduction of -5.70 mmHg [95%CI (-8.45, -2.96); p<0.0001; I^2^=62%] for systolic BP and -2.03 mmHg [95%CI (-3.84, -0.22); p<0.03; I^2^=0%] for diastolic BP, respectively (Figures 4 and 5). The level of evidence is considered low for systolic BP and moderate for diastolic, in the office.

#### Safety

**Figure 6:**
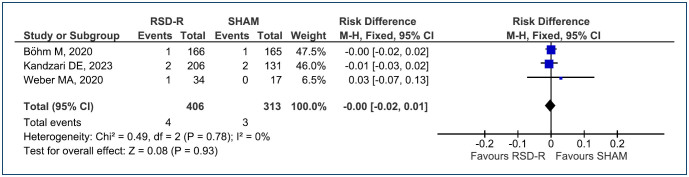
Forest plot of comparison: 1 RF-RDN versus SHAM in the change of blood pressure, outcome: 1.5 Severe Adverse Events.

The assessed composite outcome is the occurrence of severe adverse events: hypertensive crisis requiring medical attention, new stroke, and/or vascular complications (necessitating surgical repair, thrombin intervention procedure, or blood transfusion). For this outcome, three RCTs^13-15^ with a total of 719 evaluated patients were included in a follow-up of up to 6 months. In the comparison of RF-RDN with SHAM, no difference was observed between the two procedures (RD=-0.00 [95%CI -0.02, 0.01]; p=0.93; I^2^=0%) (Figure 6). The level of evidence is considered moderate.

### Evidence synthesis

#### Radiofrequency renal denervation compared with SHAM:

- Reduces, in mean, ambulatory systolic BP (24 h) at 2–3 and 6 months (-2.5 and -2.3 mmHg, with low and moderate levels of evidence, respectively).
- Reduces, in mean, ambulatory diastolic BP (24 h) at 2–3 months (-2.18 mmHg; low level of evidence) and shows no difference at 6 months (moderate level of evidence).
- Reduces, in mean, office systolic and diastolic BPs at 2–3 and 6 months (approximately -5 and 2.6 mmHg, with low and moderate levels of evidence, respectively).
- There is no difference in this comparison for serious adverse events with moderate level of evidence.

## DISCUSSION

Unlike previous meta-analyses, this analysis offers new details and information. In addition to the usual analysis for changes in office BP and changes in ambulatory BP, we conducted analyses including only the "second-generation studies," considering the various arguments against combining "first- and second-generation" RDN trials in a single meta-analysis. Among these arguments, we highlight not only the strict control of medication intake but also the improvement in all other practical methods. This includes BP measurements, patient selection, and the execution of the ablation procedure itself. These "second-generation" trials feature new catheter designs and a greater number of ablations in the arteries, thus being enhanced and distinct compared with the "first-generation trials." This meta-analysis is the first to exclusively incorporate studies assessing sympathetic denervation through radiofrequency in comparison with a placebo. Additionally, it includes comprehensive results from the SPYRAL HTN ON MED trial, constituting 46% of the total data from "second-generation" placebo-controlled trials. As a follow-up to the primary analysis, we gathered data from both the Pilot and Expansion phases. The longest follow-up time presented thus far is 6 months. In this meta-analysis, we present the results separately for the 2–3- and 6-month follow-ups.

In this review, we incorporated a study involving hypertensive patients not treated for 3–4 weeks before randomization and another study involving patients using 1–3 medications for ≥6 weeks and having uncontrolled hypertension. The systematic review conducted by Ahmad et al.^16^ assessed the impact of RDN on ambulatory and office BP in hypertensive patients. They utilized a metaregression with mixed-effects models to explore any significant interaction between the characteristics of the clinical trial and the effect size on ambulatory systolic BP.

The results of Ahmad et al.'s metaregression revealed no significant interaction between the presence of baseline antihypertensive medications and the effect size. This indicates a consistent effect size of RDN, irrespective of whether used in patients not yet on medication or in those already taking medications but with inadequate control. The difference observed was -1.10 mmHg for trials without medications (95%CI -4.40 to -2.2 mmHg; p=0.514). Our current analysis reveals a reduction in ambulatory systolic BP (24 h) at 2–3 and 6 months with RF-RDN, showing decreases of -2.5 and -2.3 mmHg, supported by low and moderate levels of evidence, respectively. Furthermore, in "second-generation" trials, RF-RDN resulted in a modest yet significant reduction in ambulatory diastolic BP (24 h) at 2–3 months (-2.18 mmHg; low level of evidence) and exhibited no significant difference at 6 months (moderate level of evidence).

Another noteworthy finding from this meta-analysis is the reduction in office systolic and diastolic BPs at 2–3 and 6 months with RF-RDN, amounting to approximately -5 and 2.6 mmHg, supported by low and moderate levels of evidence, respectively. The results suggest the safety of procedures in "second-generation" studies, showing no evidence of a difference in the occurrence of serious adverse events such as hypertensive crises requiring medical attention, new stroke, and/or vascular complications between RF-RDN and the Sham group.

### Study limitations

Our primary (intermediate) outcomes display either zero or quite acceptable heterogeneity. Nonetheless, the primary limitations of this meta-analysis encompass the small number of included RCTs (N=3), a relatively modest overall sample size (n=719 [406 randomized to RF-RDN and 313 to the control group]), and a short follow-up period (up to 6 months). Undoubtedly, more extensive and larger RCTs are warranted, providing sufficient power to yield more precise information (GRADE system level of evidence: low/moderate) regarding the role of renal sympathetic denervation by radiofrequency in treating primary hypertension and evaluating clinical outcomes.

## CONCLUSION

This meta-analysis suggests that RDN shows positive short-term results, offering a potential contribution to the enhanced management of uncontrolled hypertension in an ideal population. However, the observed effect seems relatively modest. Additionally, the results indicate that when compared with RF-RDN, the sham intervention also significantly influences BP reduction, in both office and ambulatory settings (24 h), highlighting the substantial impact of its effect. Further research is warranted to demonstrate the clinical benefits associated with this reduction.
